# Supplementary material for: Targeting the Structural Maturation Pathway of HIV-1 Reverse Transcriptase
Source: Biomolecules. 2023 Nov 1;13(11):1603. doi: 10.3390/biom13111603 (PMC10669680; doi:10.3390/biom13111603)
Supplement: Supplementary file 1 [file biomolecules-13-01603-s001.zip › biomolecules-2570032-supplementary.pdf]

Supplementary Figures and Tables

Targeting the structural maturation pathway of HIV-1 Reverse Transcriptase

By T. W. Kirby, S. A. Gabel, Eugene F. DeRose, L. Perera, J. M. Krahn, L. C. Pedersen, and R. E. London

Note: Detailed information for cited references is available in the accompanying paper.

```
RT    1                  PISPIETVPV  KLKPGMDGPK  VKQWPLTEEK  IKALVEICTE  MEKEGKISKI  GPENPYNTPV
FPC1 -9        SDHHHHHHG PISPIETVPV  KLKPGMDGPK  VKQWPLTEEK  IKALVEICTE  MEKEGKISKI  GPENPYNTPV
FPC2 -9        SDHHHHHHG PISPIETVPV  KLKPGMDGPK  VKQWPLTEEK  IKALVEICTE  MEKEGKISKI  GPENPYNTPV

RT    61       FAIKKKDSTK  WRKLVDFREL  NKRTQDFWEV  QLGIPHPAGL  KKKKSVTVLD  VGDAYFSVPL  DEDFRKYTAF
FPC1  61       FAIKKKDSTK  WRKLVDFREL  NKRTQDFWEV  QLGIPHPAGL  KKKKSVTVLD  VGDAYFSVPL  DEDFRKYTAF
FPC2  61       FAIKKKDSTK  WRKLVDFREL  NKRTQDFWEV  QLGIPHPAGL  KKKKSVTVLD  VGDAYFSVPL  DEDFRKYTAF

RT    131      TIPSINNETP  GIRYQYNVLP  QGWKGSPAIF  QSSMTKILEP  FRKQNPDIVI  YQYMDDLYVG  SDLEIGQHRT
FPC1  131      TIPSINNETP  GIRYQYNVLP  QGWKGSPAIF  QSSMTKILEP  FRKQNPDIVI  YQYMDDLYVG  SDLEIGQHRT
FPC2  131      TIPSINNETP  GIRYQYNVLP  QGWKGSPAIF  QSSMTKILEP  FRKQNPDIVI  YQYMDDLYVG  SDLEIGQHRT

                          ←Palm C-term →              ←――――――――――――――――――――――――――――――
RT    201      KIEELRQHLL  RWGLTTPDKK  HQKEPPFLWM  GYELHPDKWT  VQPIVLPEKD  SWTVNDIQKL  VGKLNWASQI
FPC1  201      KIEELRQHLL  RWGLTTPD--  ----------  GYELHPDKWT  ----------  ----------  ----------
FPC2  201      KIEELRQHLL  RWGL------  ---EPPFLWM  GYELHPDKWT  ----------  ----------  ----------

               ――――――――――――― Thumb ――――――――――――――――――――――――――――――――→
RT    271      YPGIKVRQLS  KLLRGTKALT  EVIPLTEEAE  LELAENREIL  KEPVHGVYYD  PSKDLIAEIQ  KQGQGQWTYQ
FPC1  271      ----------  ----GSGSGG  ----------  ----------  --------YD  PSKDLIAEIQ  KQGQGQWTYQ
FPC2  271      ----------  ----GSGSGG  ----------  ----------  --------YD  PSKDLIAEIQ  KQGQGQWTYQ

RT    341      IYQEPFKNLK  TGKYARMRGA  HTNDVKQLTE  AVQKITTESI  VIWGKTPKFK  LPIQKETWET  WWTEYWQATW
FPC1  341      IYQEPFKNLK  TGKYARRRGA  HTNDVKQLTE  AVQKITTESI  VIWGKTPKFK  LPIQKETWET  WWTEYWQATW
FPC2  341      IYQEPFKNLK  TGKYARRRGA  HTNDVKQLTE  AVQKITTESI  VIWGKTPKFK  LPIQKETWET  WWTEYWQATW
                       ← ΔαM →
RT    411      IPEWEFVNTP  PLVKLW
FPC1  411      IPEWEFV---  ------
FPC2  411      IPEWEFV---  ------
```

Figure S1. Design of Fingers/palm/connection constructs. FPC constructs were designed as described in the text with deletions of the disordered thumb and the C-terminal residues, and additional deletion of the palm C-terminal segment in order to block the ability of the p51 (or p66) ground state to adopt the rearranged excited state structures. In these studies, we used either the same deletion (Lys219—Met230) described in our previous studies for FPC1 [13], or an alternate deletion in the same region (Thr215 – Lys223) for FPC2 corresponding to the disordered residues in chain B of structure PDB: 5CYQ [22]. Because deletion of the thumb creates a significant gap of almost 19 Å between the terminal palm Thr[240] and connection Tyr[319] residues, an additional linking segment of sequence GSGSGG was added to connect these two residues. Met357 is a poorly conserved surface exposed residue that produces an intense resonance that tends to obscure observation of more useful resonances. Consequently, FPC1 and FPC2 were modified to include M357R mutations. The related sequences of RT (strain HBX2), FPC1, and FPC2 are shown above.

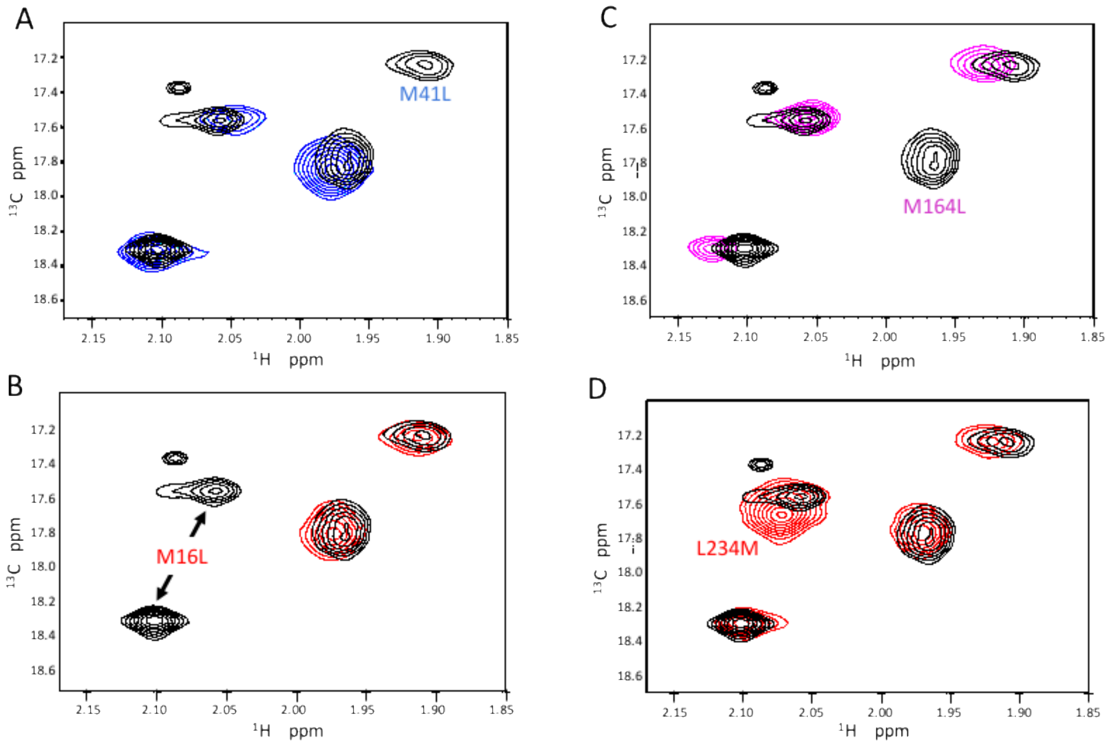

Figure S2. **Assignment of the methionine methyl resonances of FPC1.** Expanded region of the ^{1}H-^{13}C HMQC NMR spectrum containing Met16, Met41 and Met164 resonances. The spectrum of FPC1 is shown in black, and the spectra of FPC1(M41L) is shown in blue in panel A; FPC(M16L) is shown in red in panel B; FPC1 (M164L) in magenta in panel C, FPC1(L234M) is shown in red in panel D. Met230 is deleted in the FPC1 construct, and the poorly conserved and relatively uninformative Met357 was mutated to Arg. Met184 produces a weak resonance at position $\delta^{1}H,\delta^{13}C$ = 1.73, 16.66 ppm (see Figure 6), and was also assigned using an FPC1(M184L) mutant. The observation of two M16 resonances has been discussed previously, and presumably results from a slow conformational exchange process [26].

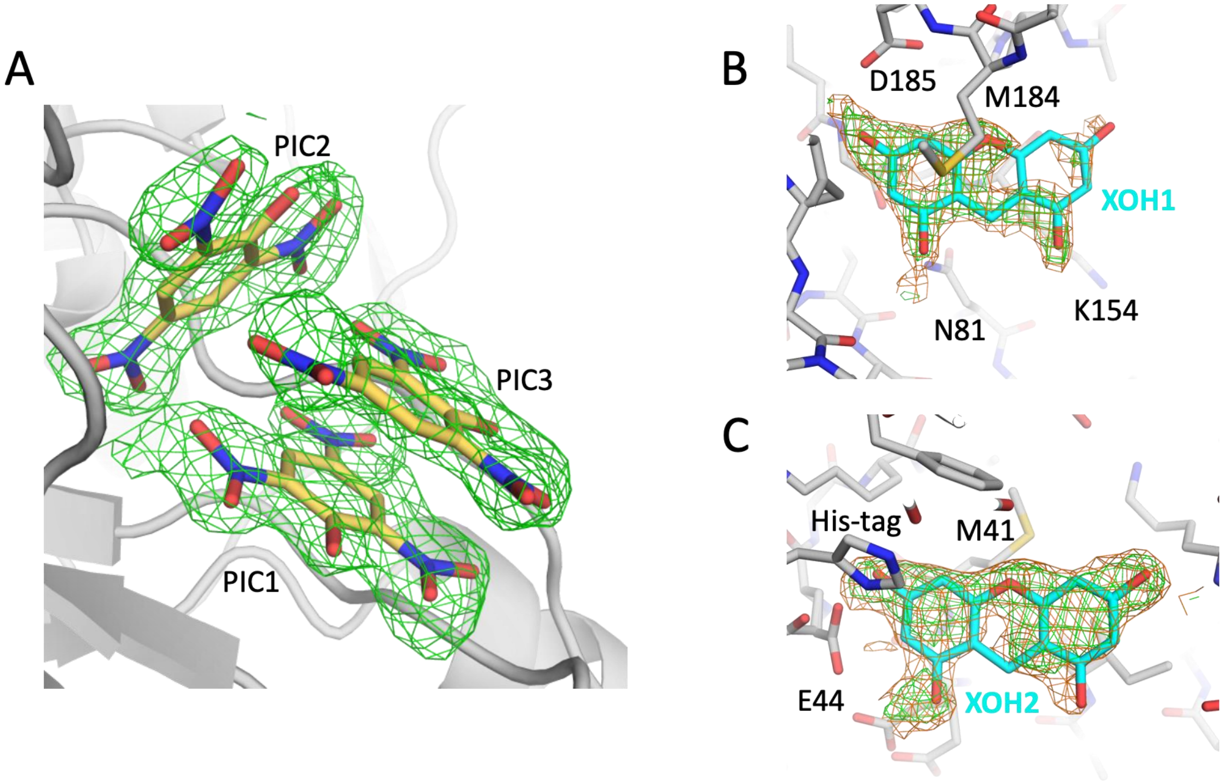

Figure S3. Fo-Fc omit maps for FPC1 complexes with picrate and Xanthene tetrol. A) Fo-Fc simulated annealing omit map (green contoured at 2.0σ) of the picrate molecules (gold) bound to the FPC1 construct. B) Fo-Fc simulated annealing omit maps of the partial occupancy 9H-xanthene-1,3,6,8-tetrol molecules (upper, XOH1; lower, XOH2) bound to the FPC1 construct. The omit maps are contoured at 2.0 σ (green) and 1.5 σ (orange).

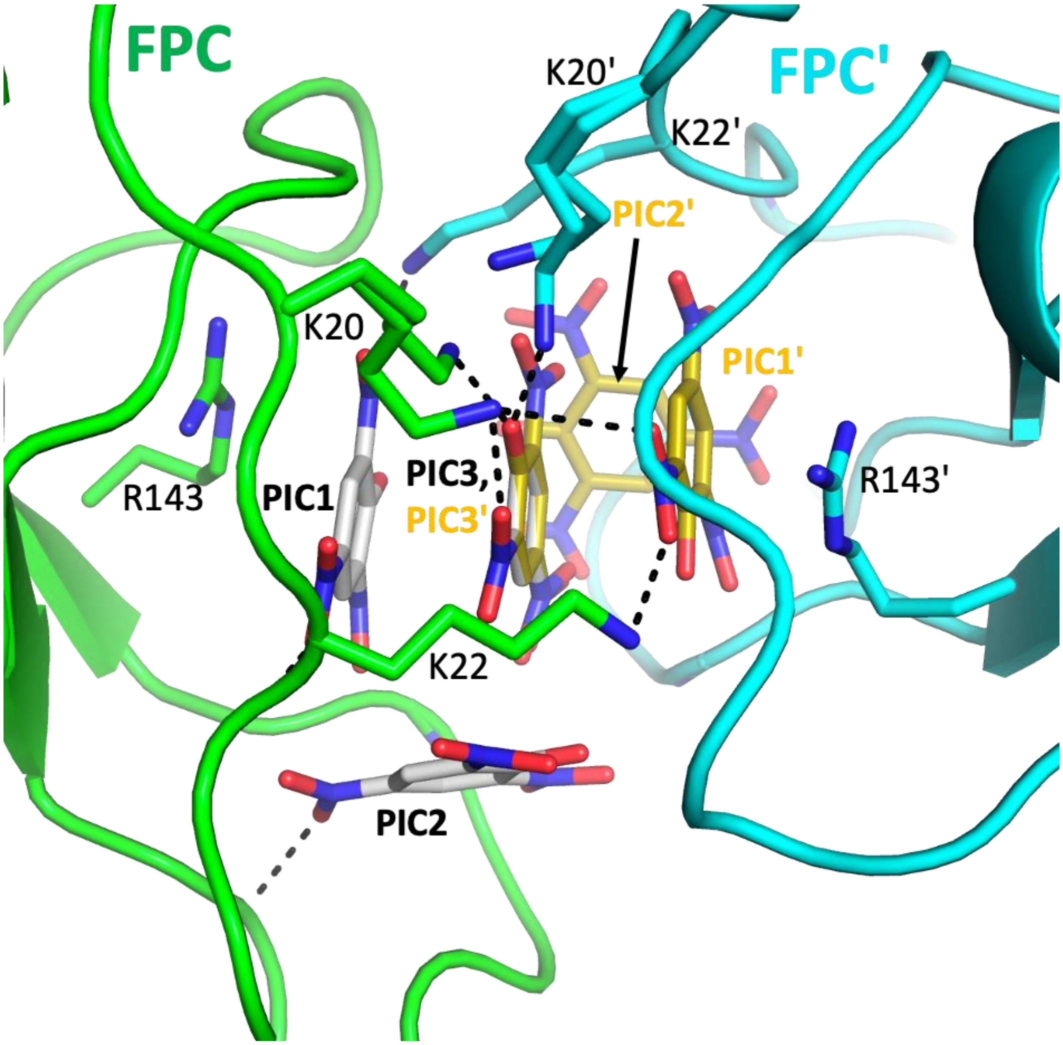

Figure S4. Picrate supports formation of an FPC lattice contact. Ribbon diagram of the FPC1-picrate complex crystallized as described in Methods showing the interface with a second FPC' molecule. The picrate cluster forms a lattice contact that mediates the interaction of two FPC molecules, with the PIC3 molecule common to both clusters. Lys20 on FPC (green) and Lys20' on FPC' (cyan) interact with the common PIC3 ligand, while Lys22 (green), forms long intermolecular H-bonds the with PIC1' (gold). As indicated in the figure, the electron density for Lys20 and Lys20' were fit to two alternate sidechain conformations. Facilitation of crystallization is supported by these direct intermolecular contacts, and probably by the stabilization of specific conformations of the two flexible loops that surround the picrate binding site.

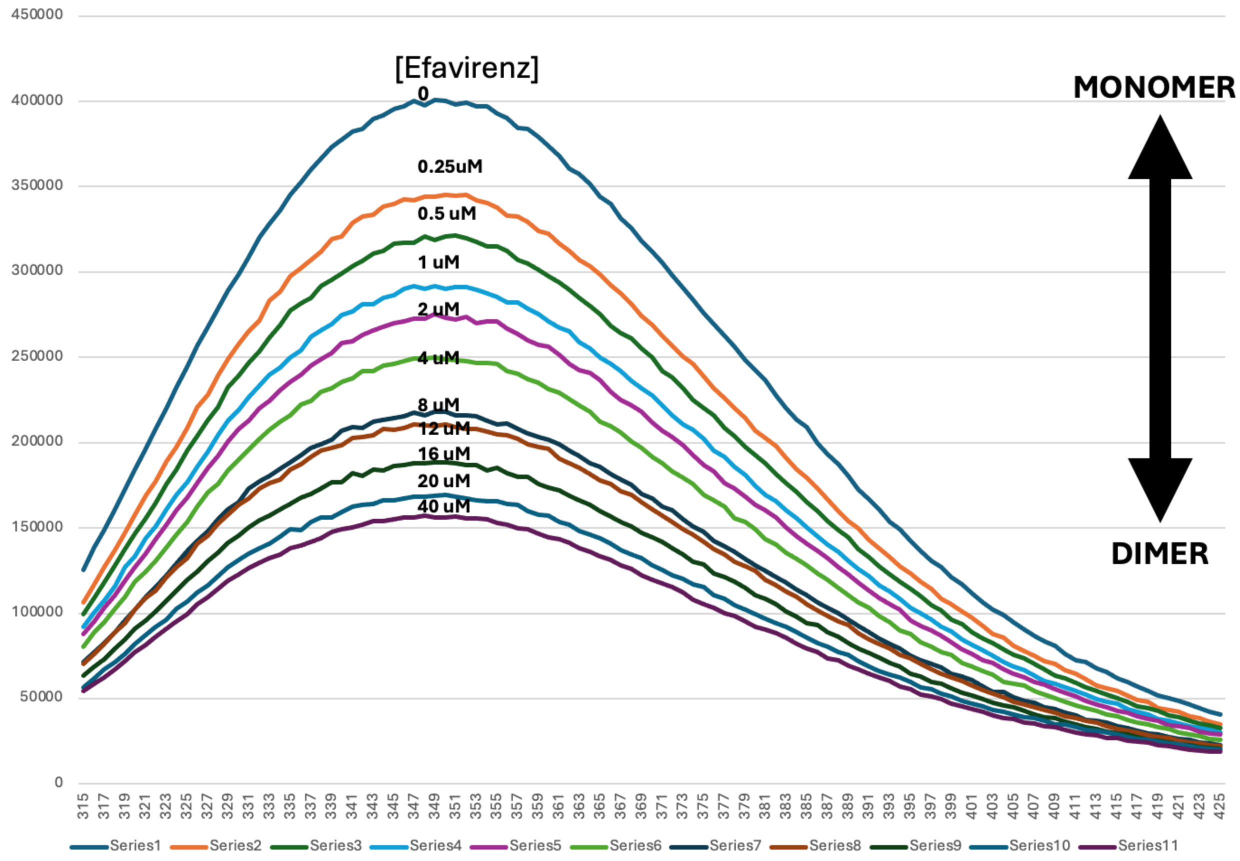

Figure S5. Intrinsic fluorescence of p51 as a function of added efavirenz. A series of fluorescence emission spectra of a sample of 2 µM p51 as a function of added EFV at the concentrations indicated. The sample was irradiated at 295 nm at 25° C using a Horiba FluoroMax fluorometer. Buffer was 50 mM KCl, 50 mM Tris, pH 8.0, 4 mM $MgCl_2$, 1 mM TCEP, 0.25 mM sodium azide, 10% DMSO. The EFV was added from a stock solution of 1 mM EFV in DMSO. Intrinsic fluorescence of p51 is quenched in response to the addition of the NNRTI efavirenz. Dimerization-dependent quenching of the intrinsic fluorescence has also been reported for p66-p51 heterodimerization [57].

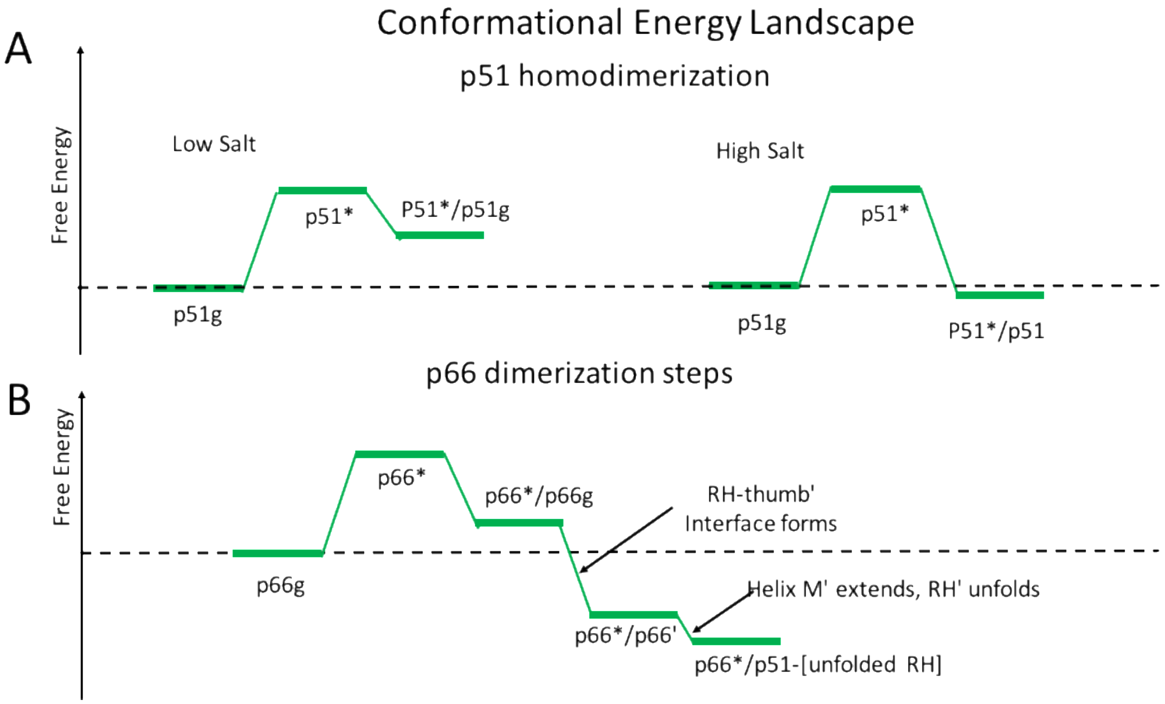

Figure S6. Stability changes associated with p51 and p66 dimerization. A) For p51 at low ionic strength, the energy gain resulting from dimer formation is significantly lower than the energy cost of forming p51*, so that little dimer is present. At high ionic strength (upper right diagram), there is sufficient stabilization of the asymmetric p51*/p51g dimer to make it readily visible by various techniques. B) For p66, the initially formed p66*/p66g homodimer is structurally similar to the p51 homodimer, with both RH and RH' domains lacking most or all interface contracts and the p66' thumb domain position variable. Subsequent formation of the RH-thumb' interface forms a p66/p66' homodimer in which helix M' is only partially formed and the RH' domain is folded but lacks additional interface contacts. Subsequent and relatively slow transfer of N-terminal RH' residues into α-helix M' destabilizes RH', leading to RH' domain unfolding and making the protease cleavage site accessible to the protease. Removal of the RH' fragment that follows the cleavage produces further RT heterodimer stabilization and makes the process irreversible. The initial formation of the asymmetric p66*/p66g homodimer is also subject to enhancement at high ionic strength (not explicitly shown above).

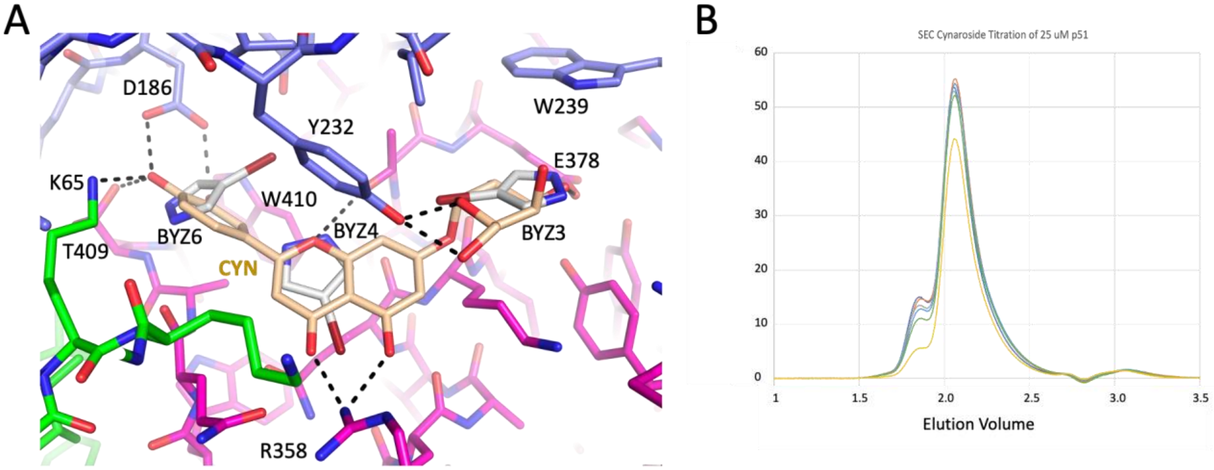

Figure S7. Prediction and SEC analysis of cynaroside binding to p51. A) OpenEye software ranked luteolin-O-glucoside (cynaroside) as the 6th highest BYX346 binding ligand in its database. As illustrated above, the glucosyl moiety overlaps with BYZ3 and the luteolin overlays BYZX4 and BYZ6. B) in the SEC titration study, the cynaroside concentrations were 0 (blue), 0.5 mM (red), 0.625 mM (gray), 2.0 mM (light blue), 4.0 mM (green), and 8.0 mM (yellow). These values corresponded to an IC50 = 7.6 mM.

Table S1: Data collection and refinement statistics

	Apo FPC1	Picrate	Picrate+Xanthene-1,3,6,8-tetrol	Apo FPC2
PDB ID code	8TCK	8TCL	8TCM	8TCJ
**Data collection**				
Space group	$P2_12_12_1$	I222	I222	$P3_121$
Cell dimensions				
a, *b*, *c* (Å)	53.74, 63.95, 128.30	77.579, 99.624, 104.161	77.309, 99.406, 104.747	105.080, 105.080, 54.531
α, β, γ (°)	90, 90, 90	90, 90, 90	90, 90, 90	90, 90, 120
Resolution (Å)	50.00-2.20 (2.24-2.20)[a]	50.0-1.95 (1.98 -1.95)	50.0-1.82 (1.85-1.82)	50.0-1.85 (1.88-1.85)
R_{sym} (%)	7.0 (41.3)	4.8 (38.6)	3.8 (36.0)	3.8 (81.4)
R_{meas} (%)	7.3 (44.8)	5.4 (47.3)	4.1 (45.1)	3.9 (89.4)
R_{pim} (%)	2.1 (16.5)	2.4 (26.8)	1.6 (26.6)	1.0 (36.7)
$CC_{1/2}$	0.999 (0.901)	0.999 (0.856)	1.000 (0.885)	1.000 (0.876)
I / σI	9.5 (2.1)	10.7 (1.9)	15.1 (1.6)	24.3 (2.2)
Completeness (%)	98.8 (86.1)	96.1 (84.1)	98.7 (81.2)	100.0 (100.0)
Redundancy	11.8 (5.9)	4.2 (2.5)	6.2 (2.4)	11.2 (5.8)
**Refinement**				
Resolution (Å)	25.26 - 2.20	61.62 -1.95	22.52 - 1.82	46.78 - 1.85
No. reflections	22,806	28,558	35,973	29,756
R_{work} / R_{free} (%)	20.75/ 23.96	19.82/ 22.88	19.35/ 22.30	21.58/ 25.23
CC_{work}/CC_{free}	0.956/ 0.945	0.952/ 0.959	0.951/ 0.944	0.951/ 0.922
No. atoms				
Protein	2608	2513	2532	2471
Water	55	148	196	92
Other	-	48	84	-
B-factors				
Protein	51.87	39.75	37.35	47.84
Water	48.22	38.34	41.31	47.58
Other	-	30.53	35.89	
R.m.s. deviations				
Bond lengths (Å)	0.006	0.011	0.014	0.016
Bond angles (°)	0.817	1.160	1.206	1.257
Ramachandran Plot				
Allowed (%)	2.88	2.29	1.99	2.02
Favored (%)	97.12	97.71	98.01	97.64

[a]Values in parentheses are for highest-resolution shell.

Table S2. - Structural Consistency of FPC constructs with RT, chain B and with the p51 chain B monomer[a.]

	RMSD Values comparing FPC constructs with previous structures			
RT structure	Construct evaluated			
	FPC1 apo	FPC2	FPC1+picrate	FPC1+picrate+Xanthene
PDB code	8TCK	8TCJ	8TCL	8TCM
1DO, chain B	0.723 Å<br>2091 atoms	0.487 Å<br>1995 atoms	1.346 Å<br>2195 atoms	1.329 Å<br>2159 atoms
5CYQ, chain B	0.627 Å<br>2112 atoms	0.627 Å<br>2121 atoms	1.089 Å<br>2108 atoms	1.110 Å<br>2079 atoms
4KSE monomer	0.609 Å<br>2013 atoms	0.667 Å<br>2023 atoms	1.058 Å<br>2137 atoms	1.095 Å<br>2136 atoms

[a]Evaluated based on PyMol alignments

Table S3. Software-identified Ligands obtained from NCI that were evaluated.

NSC Identifier	CAS #	OpenEye Score	rank	Chemical name
BZZ7 cavity				
725600	142434-80-8	-10.67	6	3-phosphonomethyl-D-phenylalanine
312628	66023-94-7	-10.38	8	
35122		-10.30	12	
407331		-10.29	13	robinetin
59266	93432-80-5	-10.24	16	3, 3',4',5',7-pentahydroxy flavonone
143541	4371-21-5	-10.24	18	3,3',4,4',5,5'-Biphenylhexol
24655		-10.21	19	
76429		-9.94	32	
164021		-9.88	33	
164020	10444-59-4	-9.85	35	
28120	3569-77-5	-9.65	39	Amidapsone
367044	81613-36-7	-9.64	40	
120804		-9.64	41	
305784	51123-89-8	-9.62	42	
BYZ34 cavity				
101563	17415-78-0	-15.22	2	
204542		-13.69	10	
139603		-13.34	14	
294205		-13.09	22	
369503		-13.04	23	N-(p-coumaroyl)-serotonin
115998		-12.78	33	
157496		-12.67	38	
204574		-12.65	41	
81508		-12.49	55	
354964		-12.46	58	
303243	84396-05-4	-12.43	61	
BYZ46 cavity				
350081		-15.08	1	9H-xanthene-1,3,6,8-tetrol
141926	66907-77-5	-14.71	5	
339348	74377-94-9	-14.14	12	
619690		-14.09	13	
BYZ346 cavity				

350081		-15.08	2	9H-xanthene-1,3,6,8-tetrol
210469		-14.97	4	
403426	7404-05-9	-14.89	5	5-(oxan-4-yl)-5-phenylimidazolidine-2,4-dione (phenytoin analog)
700145		-14.82	6	Luteolin-7-O-β-D-glucoside; cynaroside
212367	21333-05-1	-14.06	17	
619690		-13.80	27	
210585		-13.70	30	
135821	40496-49-9	-13.42	51	
E399 binding cavity				
47715		-12.96	2	Mordant Black 3; alizarine blue OCBN
299391	3147-14-6	-11.98	10	calmagite
40932				stilbene-4,4'-dicarboxylate

The NSC number is a compound identifier assigned by the Developmental Therapeutics Program (DTP) of the NCI. NSC is an abbreviation for Cancer Chemotherapy National Service Center. The OpenEye score and the rank for binding to the cavity evaluated are also indicated. Extensive information about each compound is available by entering the NSC# in the PubChem web site: https://pubchem.ncbi.nlm.nih.gov/ In some cases we have also provided the CAS# and the chemical names. As noted in the text, many of the highest scoring ligands were unavailable from the depository. Since most of the high affinity BYZ46 ligands also bind to BYZ346 cavity, there are fewer entries for this site. In addition to limitations imposed by compound availability, in some cases we selected lower ranking ligands in order to obtain greater chemical diversity. In the case of NSC350081, we listed both the BYZ46 and BYZ346 scores to indicate that there was some difference in scoring. Although not apparent from this abbreviated list, some structural groups including hydroxylated biphenyls, flavonoids and phenytoin analogs appeared numerous times in the complete lists.
